# Anticancer and anticholesterol attributes of sea cucumbers: An opinion in terms of functional food applications

**DOI:** 10.3389/fnut.2022.986986

**Published:** 2022-08-04

**Authors:** Netty Salindeho, Fahrul Nurkolis, William Ben Gunawan, Matthew Nathaniel Handoko, Mrinal Samtiya, Rendy Dijaya Muliadi

**Affiliations:** ^1^Fishery Products Technology Study Program, Faculty of Fisheries and Marine Sciences, Sam Ratulangi University, Manado, Indonesia; ^2^Biological Sciences, State Islamic University of Sunan Kalijaga (UIN Sunan Kalijaga), Yogyakarta, Indonesia; ^3^Nutrition Science Department, Faculty of Medicine, Diponegoro University, Semarang, Indonesia; ^4^Department of Nutrition Biology, Central University of Haryana, Mahendragarh, India; ^5^Health and Nutrition Science Executive, Nutrifood Research Center, PT Nutrifood Indonesia, Kawasan Industri Pulogadung, Jakarta, Indonesia

**Keywords:** functional food, sea cucumbers, cancer, cholesterol, marine products

## Introduction

Sea cucumbers, marine invertebrates belonging to the phylum Echinodermata and specifically to the class Holothuroidea, are fishery products that possess a high economic value and potent medicinal properties (1, 2). In terms of physical appearance, sea cucumbers are tube-like marine animals with skin that resembles a leathery material (2). As of late, research has been conducted on sea cucumbers, especially on their role as a functional food. Sea cucumbers, especially *Actinopyga mauritiana*, contain a high percentage of protein or peptides (63.3 ± 0.43%), with glycine as its most abundant amino acid. *A. mauritiana* also possesses a low lysine:arginine ratio. Both properties are shown to exert hypocholesterolemic effects (3). Sea cucumbers also contain certain proteins (bioactive peptides), polysaccharides, and saponins, and when extracted, these compounds are shown to exert anticancer effects (4, 5). Saponins from sea cucumbers promote osteoblast differentiation or osteogenic differentiation from pre-osteoblasts by activating molecular pathways of BMP2/Smads activation in MC3T3-E1 cells (6). More interestingly, sulfated polysaccharides in sea cucumber have the potential to be used as health-improving agents and processed through technology and combining (7), such as into functional food. Sea cucumber is a potential functional food based on the medicinal properties mentioned above. Hence, this article's main aim is to interpret the latest findings on potential applications of sea cucumbers as a functional food.

## Sea cucumbers in general

Sea cucumbers, also known as *teripang, “trepan,”* or“*beche-de-mer,”* are marine invertebrates that belong to the phylum Echinodermata and the class Holothuroidea. Sea cucumbers can be found in deep seas and have a long history of being used as food and medicine in the Middle East and Asia (1). It possesses a tube-like soft body with leathery skin. The shape of sea cucumbers resembles that of a cucumber, hence the name (1, 3). Currently, there are about 1,716 species of sea cucumbers in the world, and the most biodiverse of sea cucumbers are located in the Asia Pacific region (3). Some commonly consumed sea cucumbers that possess health benefits are *Holothuria leucospilota, Bohadschia argus, Pearsonothuria graeffei, Holothuria polii, Colochirusancep, Holothuria arenicola, Cucumaria japonica*, and several others (4). Sea cucumbers contain numerous bioactive compounds such as chondroitin sulfates, saponins, peptides, and glycosaminoglycan, and these compounds possess anticoagulation, antiangiogenesis, anticancer, antidiabetic, and antibacterial activities (5). Besides, sea cucumbers also contain micronutrients such as thiamine or vitamin B1, B2 or riboflavin, B3 (niacin), vitamin A (retinol, retinyl esters), calcium, iron, zinc, and magnesium (4).

## Anticancer property of sea cucumbers

**Figure 1:**
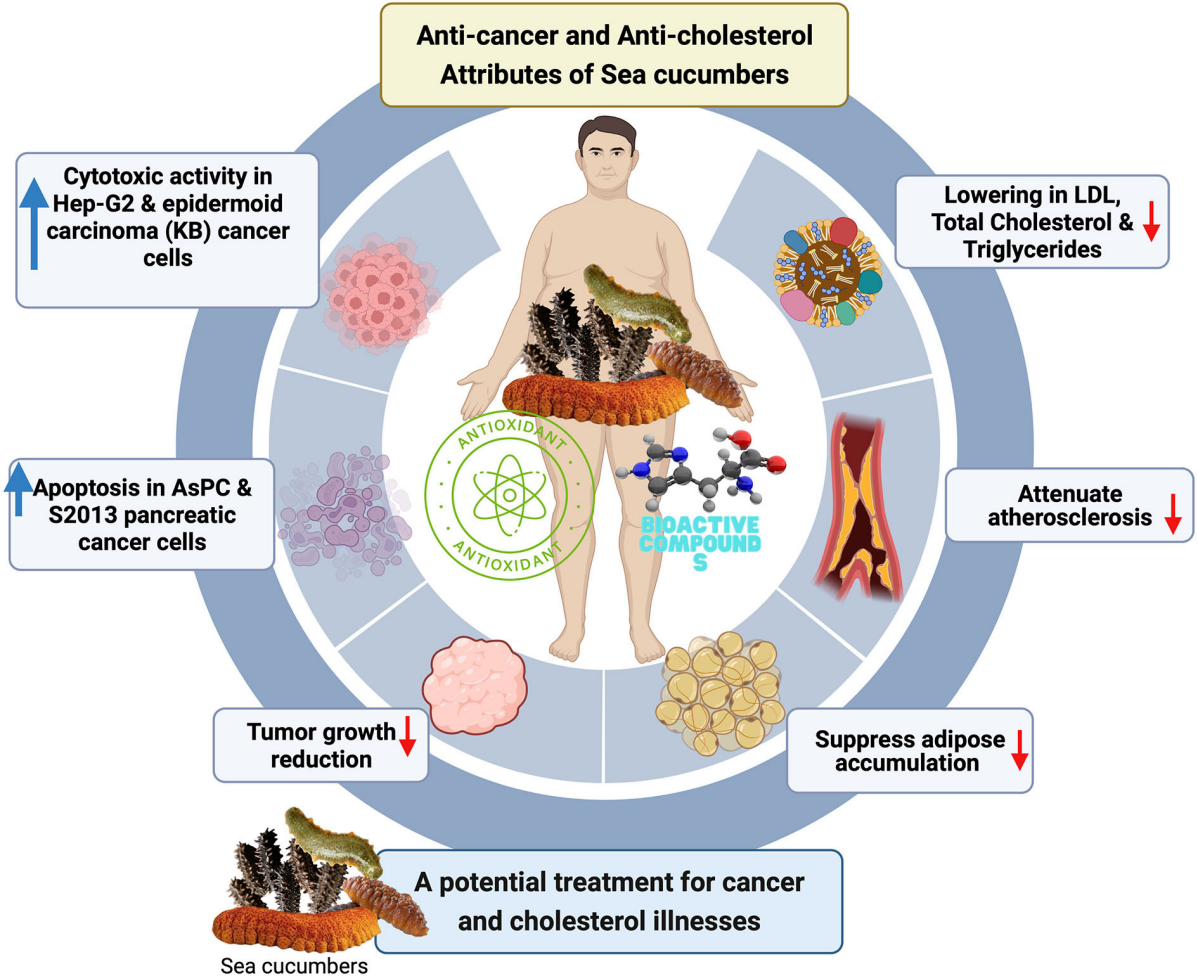
Sea cucumbers effect possible-scheme for anti-cancer and anti-cholesterol.

As mentioned previously, sea cucumbers exhibits an anticancer activity. Anticancer activities can be achieved through cytotoxic activity, apoptosis induction, cell cycle arrest, tumor growth reduction, metastasis inhibition, and drug resistance inhibition methods (Figure 1) (4). Cytotoxic activity is achieved by blockage or growth prevention of cancer cells. *Holothuria scabra* species produce bioactive carbohydrate compounds such as holothurine A3 and A4 that were shown to be cytotoxic in hepatocellular carcinoma (Hep-G2) and epidermoid carcinoma (KB) cell lines (8). In the apoptosis induction aspect, Frondanol A5, a compound obtained from *Cucumaria frondosa* extract, caused apoptosis in S2013 and AsPC pancreatic cancer cells (9). Cell cycle arrest is also a potential mechanism for inhibiting cancer cell growth. *Pearsonothuria graeffei* contains Ds-echinoside A and Echonoside A that disrupt the G0/G1 cell cycle process of liver carcinoma cells (Hep-G2), disrupting the preparation for DNA replication (10). *Pentacta quadrangulari* possesses a tumor growth reduction ability because of its saponin content, specifically Philinopsides E and A in sarcoma 180 and hepatoma 22 mouse models (11, 12). *P. graeffei* contains Ds-echinoside A, which prevents cell migration and invasion, and adhesion of hepatocellular carcinoma (Hep-G2) cells, hence reducing the probability of metastasis (development of new cancer site) of cancer cells (13). Cancer cells could develop drug resistance; hence drug resistance inhibition is crucial in chemotherapy (4). *Cucumariaokhotensis* possesses a type of saponin named Frondoside A that inhibits autophagy to survive in human urothelial carcinoma cell lines. Hence, cancer drug resistance is inhibited (14).

## Anticholesterol property of sea cucumbers

Diverse sea cucumbers have demonstrated that they could be an intriguing natural source of useful substances because they contain amino acids, vitamins, triterpene glycosides, PUFAs, flavonoids, polysaccharides, carotenoids, minerals, collagen, gelatin, phenolic, saponin, and bioactive peptides (3, 15). From a nutritional standpoint, sea cucumber is the perfect food with medical significance, since it includes more proteins and less fats than most other foods. Bioactive compounds and pigments in sea cucumbers exhibit an antioxidant activity, which may have the potential to improve cholesterol and reduce the possibility of atherosclerosis using many capable mechanisms (Figure 1) (16). Polysaccharides from *Apostichopus japonicus* also have antihyperlipidemic and antioxidant attributes (17). Triterpene glycosides (or triterpene saponin) exhibit hypolipidemic (18), anti-inflammatory (19), immunomodulatory (20), and wound-healing properties (21), which indirectly contribute to dyslipidemia. Triterpene glycosides are also known to interact with sterol in the membrane of sea cucumbers as a self-defense mechanism against saponin (22, 23). In obese mouse models, sterol sulfate significantly reduced insulin resistance with inflammation induced by high-fat, high-fructose diets (24). The saponin from sea cucumbers has been proved to suppress adipose accumulation (Figure 1) (25), lower lipid levels, and attenuate atherosclerosis (26). Saponin can also modulate cholesterol metabolism in *Thelenota ananas* (27). These facts stated the health-related beneficial effects of the both saponin and the sterol in sea cucumbers.

The lipid content of *Australostichopus mollis* consists of high levels of 54% PUFA compared to MUFA (23%) and SFA (24%), with arachidonic acid followed by eicosapentaenoic acid as the dominant PUFA (28). Replacing SFAs with PUFAs was known to reduce total cholesterol levels and provide beneficial cardio-metabolic effects (29–31). Wen et al. (32) also found that arachidonic acid was the dominant composition of PUFAs in many species of sea cucumbers. They further elaborated that while fatty acid profiles varied between species, amino acid levels were comparable. These facts suggest the potential of sea cucumbers as a high-protein source with low total fat and high unsaturated fatty acid contents. The sea cucumber powder (33) and dietary glucosylceramide from sea cucumber (34) also significantly decreased the cholesterol level in mice models fed with high fat-enriched diet.

## Future functional food product development of sea cucumbers

Significant efforts have been made to identify more therapeutic-related food and their pharmaceutical applications, along with the growing understanding of the health-beneficial properties of compounds derived from sea cucumbers. The numerous therapeutic benefits of sea cucumbers and their valuable bioactive components have shown their potential as both functional meals and a natural source of novel multifunctional medications (35). However, despite high hopes, only a few foodstuffs made from sea cucumbers are available in the food and medical industries now. One of the techniques to utilize sea cucumbers as a functional food product would be to fortify food and health products with sea cucumbers to increase the customer acceptability of sea cucumbers effectively. Moreover, depending on seasonal fluctuations, geographic location, and feeding practices, sea cucumbers' proximate composition varies significantly, resulting in sea cucumbers being versatile food ingredients to be utilized for a specific health benefit or personalized nutrition (15). Xu et al. (36) also emphasized that the potential for creating high-quality nutraceutical products by extraction and purification of bioactive chemicals found in sea cucumbers has not been completely explored.

## Discussions

The morbidity due to cancer has increased daily in both developed and developing nations, and it is one of the major causes of death, as nearly ten million deaths are caused by cancer every year worldwide (37). Another illness condition is high body cholesterol, which also affects people universally and may be a primary cause of several disorders such as atherosclerosis, and cardiovascular diseases. Therefore, there is an urgent need for a remedy that can be used to treat these ailments. Marine organisms are augmented for development of drugs; about 10% of these creatures' extracts contain anticancer attributes. In addition, the organisms' extracts are categorized by reduced drug resistance, lower toxicity, safety, and high efficiency (5). Sea cucumber has been explored for its anticancer and cholesterol-lowering activities due to the presence of potential bioactive components (Figure 1). For instance, sea cucumber (golden) contains several bioactive components such as saponin, flavonoids, docosahexaenoic and eicosapentaenoic acid (EPA-DHA), proteoglycans, mucopolysaccharide, heparin sulfate, heparin, dermatan sulfate, chondroitin sulfate, hyaluronic acid, glycosaminoglycan, collagen, and glycoprotein and has several promising attributes beyond its nutritional qualities (38–40). In conclusion, the opinion suggested that sea cucumber and its derived bioactive peptide and carbohydrate components could be a point of great interest for future research as a potential treatment for cancer and cholesterol illnesses (Figure 1). It is just an opinion that summarizes sea cucumber's potential as a new hope for these ailments, but for validation of these health benefits, there is a need to perform extensive studies (especially *in vivo* and clinical trials); after that, it may be further considered for development of functional foods or nutraceuticals.

## Author contributions

NS, FN, MH, and WG contributed to the conception and design of the opinion-study and drafted the manuscript first. MS, FN, and RM edited, revised, and approved the final version of the submitted manuscript. All authors contributed to the article and approved the submitted version.

## Conflict of interest

The authors declare that the research was conducted in the absence of any commercial or financial relationships that could be construed as a potential conflict of interest.

## Publisher's note

All claims expressed in this article are solely those of the authors and do not necessarily represent those of their affiliated organizations, or those of the publisher, the editors and the reviewers. Any product that may be evaluated in this article, or claim that may be made by its manufacturer, is not guaranteed or endorsed by the publisher.

## Abbreviations

DNA: deoxyribonucleic acid
PUFAs: polyunsaturated fatty acids
MUFAs: monounsaturated fatty acids
SFAs: saturated fatty acids
EPA: eicosapentaenoic acid
DHA: docosahexaenoic acid.
